# Expression of β-Catenin in Salivary Gland Tumors

**DOI:** 10.7759/cureus.72249

**Published:** 2024-10-24

**Authors:** Hira Batool, Faria W Khan, Azra Bashir, Zubaria Rafique, Bahar E Mustafa, Kanwal Babar, Akhtar Sohail Chughtai, Aribah Atiq

**Affiliations:** 1 Histopathology, Chughtai Institute of Pathology, Lahore, PAK; 2 Infectious Disease, University of Melbourne, Melbourne, AUS

**Keywords:** head and neck neoplasm, salivary gland tumors, warthin tumor, wnt signaling pathway, β-catenin

## Abstract

Introduction: Salivary gland tumors are an important group of neoplasms in the head and neck region. This study aims to assess the significance of β-catenin expression in both benign and malignant salivary gland tumors.

Materials and methods: We included 80 reported cases of benign and malignant salivary gland tumors and employed β-catenin stain on tumor blocks. A consultant histopathologist interpreted the β-catenin expression, and a score of 0, 1, 2, 3 was given based on intensity as completely absent, mild, moderate, or intense. Intracellular localization of β-catenin stain was interpreted as the percentage of membranous, cytoplasmic, or nuclear expression.

Results: Expression in benign and malignant classes of salivary gland tumors differs in intensity and localization. The benign category of tumors exhibited primarily membranous expression, and all cases of Warthin tumor showed intense membranous expression (p ≤ 0.05). Malignant tumors manifested chiefly cytoplasmic expression, and among the malignant category, adenoid cystic carcinoma showed intense cytoplasmic localization (p ≤ 0.05). None of the tumors showed nuclear expression.

Conclusion: Decreased membranous and increased cytoplasmic expression could predict malignant behavior and invasive potential.

## Introduction

Salivary gland tumors have an incidence of about 3-10% among neoplasms of the head and neck region [1]. The most common benign salivary gland tumor is pleomorphic adenoma (PA), while the most common malignant ones include mucoepidermoid carcinoma (MEC) and adenoid cystic carcinoma (ACCA) [1,2]. β-catenin is a crucial part of the cadherin-catenin complex in epithelial cells. Thus, it plays a significant role in the cohesion and divergence of epithelial structures [3]. Another acknowledged association of β-catenin is with the Wnt signaling pathway. Dysregulation of the Wnt cascade shifts the extracellular β-catenin towards intracellular location into cytoplasmic and nuclear compartments [4]. Increased nuclear β-catenin levels promote tumor progression by inhibiting T-cell responses via bonding with T-cell factors (TCFs) and accumulating oncogenes such as c-Myc and cyclin D1 [5,6].

A modified β-catenin intracellular proportion may play a role in the characteristic incohesive and invasive nature of neoplastic cells [7]. This happens due to alteration in the cadherin-catenin complex and cell signaling pathways. Thus, β-catenin has been linked to carcinomas of the head and neck, pancreas, lung, gastrointestinal tract, and ovaries [6].

This study aims to evaluate the expression of β-catenin immunohistochemical stain in benign and malignant salivary gland neoplasms to determine the role played by the cadherin-catenin complex in dissociation and altered differentiation of these neoplasms.

## Materials and methods

This descriptive study was conducted in the Histopathology Department of the Chughtai Institute of Pathology. Approval was obtained from the Institutional Review Board (CIP/IRB/1194). Data for this study were retrieved by selecting 80 cases of salivary gland tumors over two years (July 2021-June 2023). Forty cases belonging to the benign group of tumors, with a mean age group of 40.9 years (26 men, 14 women), and 40 malignant cases, with a mean age group of 44.65 years (24 men, 16 women), were included.

β-Catenin immunohistochemistry was applied on formalin-fixed paraffin-embedded tumor blocks. The first preparation step included cutting the paraffin block at 3 µm thickness; then, these sections were taken on a slide. Slides were dried on a hot oven plate at 70°C. Next slides were put in an Autostainer, paraffin wax from the sections was removed, and the tissue was rehydrated by exposing the slides to xylene and decreasing alcohol concentration. Next, antigen retrieval was done by putting the slides in retrieval solution at 97°C for 20 min. The activity of peroxidase was inhibited with the help of a blocking solution.

The primary β-catenin antibody (DAKO, FLEX monoclonal mouse anti-human β-catenin clone) was employed; it was ready to use and prediluted. The incubation time for the primary antibody was 30 min. Finally, an insoluble chromogen compound named diaminobenzidine (DAB) was added for 10 min to detect the primary antibody, and then a counterstain named hematoxylin was applied.

External control was added to every batch of immunomarkers to ensure the quality and performance of primary antibodies. An expert consultant histopathologist identified the intensity and area of expression of antibodies, whether membranous, cytoplasmic, or nuclear, along with the proportion of each staining pattern. Statistical analysis was done using IBM SPSS Statistics for Windows, Version 23.0 (Released 2015; IBM Corp., Armonk, New York, United States).

## Results

The intensity of membranous, cytoplasmic, and nuclear staining was categorized based on a histological point scale as follows: absent (score 0, 0% expression in tumor cells), mild (score 1, 1-25% tumor cells), moderate (score 2, 26-50% tumor cells), and intense (score 3, >50% tumor cells) [5]. The mean intensity of membranous staining and the mean percentage of membranous staining of each malignant tumor in this study were compared with PA by applying an independent sample t-test and using the Mann-Whitney U test. The statistical significance of membranous pattern intensity and percentage of staining of PA was compared with salivary duct carcinoma (SDCA), MEC, ACCA, carcinoma ex pleomorphic adenoma (CA ex PA), and secretory carcinoma (SCA), and it turned out to be significant (p ≤ 0.05). Intense membranous staining was seen in Warthin tumor (WT), followed by PA, compared to other benign and malignant tumors in this study.

Although cytoplasmic staining of β-catenin is less consequential than membranous staining, in our study, we analyzed and compared the means of cytoplasmic intensity and percentage of cytoplasmic staining of PA with SDCA, MEC, acinic cell carcinoma (ACC), CA ex PA, and ACCA. Results were statistically significant (p ≤ 0.05). So, cytoplasmic staining was observed more in malignant tumors, and membranous staining was seen more in benign tumors. None of the tumor included in this study showed nuclear expression. WT showed the most intense staining among benign tumors, followed by PA. In malignant tumors, ACCA showed intense staining compared to other salivary gland tumors (Table 1).

**Table 1:** Staining intensity of β-catenin immunohistochemistry in both benign and malignant categories. PA, pleomorphic adenoma; WT, Warthin tumor; BCA, basal cell adenoma; CA, canalicular adenoma; SDCA, salivary duct carcinoma; CA ex PA, carcinoma ex pleomorphic adenoma; MEC, mucoepidermoid carcinoma; ACC, acinic cell carcinoma; ACCA, adenoid cystic carcinoma; SCA, secretory carcinoma.

		Intensity of staining			
Diagnosis	Total number of cases (n)	Absent (0)	Mild (1)	Moderate (2)	Intense (3)
Benign tumors	40/80 (50%)				
PA	22/40 (55%)	0	2	11	9
WT	14/40 (35%)	0	0	0	14
BCA	3/40 (7.5%)	0	0	2	1
CA	1/40 (2.5%)	0	1	0	0
Malignant tumors	40/80 (50%)				
SDCA	11/40 (27.5%)	0	7	3	1
CA ex PA	5/40 (12.5%)	0	0	5	0
MEC	12/40 (30%)	0	7	5	0
ACC	2/40 (5%)	0	0	2	0
ACCA	8/40 (20%)	0	1	4	3
SCA	2/40 (5%)	0	1	1	0

Benign tumors showed membranous staining chiefly, and malignant tumors depicted cytoplasmic staining, most displayed by ACCA and MEC (Table 2).

**Table 2:** Localization of β-catenin expression immunohistochemistry in both benign and malignant categories. PA, pleomorphic adenoma; WT, Warthin tumor; BCA, basal cell adenoma; CA, canalicular adenoma; SDCA, salivary duct carcinoma; CA ex PA, carcinoma ex pleomorphic adenoma; MEC, mucoepidermoid carcinoma; ACC, acinic cell carcinoma; ACCA, adenoid cystic carcinoma; SCA, secretory carcinoma.

		Cellular localization of β-catenin			
Diagnosis	Total number of cases (n)	Membranous	Cytoplasmic	Nuclear	Membranous and cytoplasmic
Benign tumors	40/80 (50%)				
PA	22/40 (55%)	20	0	0	2
WT	14/40 (35%)	14	0	0	0
BCA	3/40 (7.5%)	2	0	0	1
CA	1/40 (2.5%)	0	0	0	1
Malignant tumors	40/80 (50%)				
SDCA	11/40 (27.5%)	1	4	0	6
CA ex PA	5/40 (12.5%)	0	2	0	3
MEC	12/40 (30%)	0	10	0	2
ACC	2/40 (5%)	0	0	0	2
ACCA	8/40 (20%)	0	7	0	1
SCA	2/40 (5%)	0	2	0	0

WT showed the peak membranous staining intensity and percentage among benign tumors, while ACC showed the highest membranous intensity and percentage among malignant tumors (Table 3).

**Table 3:** Calculation of mean of intensity and percentage of membranous β-catenin expression. PA, pleomorphic adenoma; WT, Warthin tumor; BCA, basal cell adenoma; CA, canalicular adenoma; SDCA, salivary duct carcinoma; CA ex PA, carcinoma ex pleomorphic adenoma; MEC, mucoepidermoid carcinoma; ACC, acinic cell carcinoma; ACCA, adenoid cystic carcinoma; SCA, secretory carcinoma.

Diagnosis	Total number of cases (n)	Mean of membranous staining intensity	Mean of percentage of membranous staining
Benign tumors	40/80 (50%)		
PA	22/40 (55%)	2.3182	71.8182
WT	14/40 (35%)	2.9286	92.8571
BCA	3/40 (7.5%)	2.3333	76.6667
CA	1/40 (2.5%)	1.0000	80.0000
Malignant tumors	40/80 (50%)		
MEC	11/40 (27.5%)	0.2500	7.5000
CA ex PA	5/40 (12.5%)	0.8000	12.0000
ACC	12/40 (30%)	2.0000	40.0000
ACCA	2/40 (5%)	0.3750	2.5000
SDCA	8/40 (20%)	1.0000	22.7273
SCA	2/40 (5%)	0.0000	0.0000

Canalicular adenoma in benign category showed the most incredible cytoplasmic intensity and percentage, while among malignant tumors, CA ex PA and ACCA exhibited maximum intensity and percentage (Table 4).

**Table 4:** Calculation of mean of intensity and percentage of cytoplasmic β-catenin expression. PA, pleomorphic adenoma; WT, Warthin tumor; BCA, basal cell adenoma; CA, canalicular adenoma; SDCA, salivary duct carcinoma; CA ex PA, carcinoma ex pleomorphic adenoma; MEC, mucoepidermoid carcinoma; ACC, acinic cell carcinoma; ACCA, adenoid cystic carcinoma; SCA, secretory carcinoma.

Diagnosis	Total number of cases (n)	Mean of cytoplasmic staining intensity	Mean of percentage of cytoplasmic staining
Benign tumors	40/80 (50%)		
PA	22/40 (55%)	0.1364	4.0909
WT	14/40 (35%)	0.0000	5.0000
BCA	3/40 (7.5%)	0.6667	6.6667
CA	1/40 (2.5%)	2.0000	10.0000
Malignant tumors	40/80 (50%)		
MEC	11/40 (27.5%)	1.2500	24.1667
CA ex PA	5/40 (12.5%)	2.0000	74.0000
ACC	12/40 (30%)	2.0000	55.0000
ACCA	2/40 (5%)	2.2500	66.2500
SDCA	8/40 (20%)	1.3636	52.7273
SCA	2/40 (5%)	1.5000	60.0000

## Discussion

β-catenin is an essential component of the cadherin-catenin complex, forming tight junctions between cells, and its expression by immunohistochemistry is membranous. As tumors acquire invasive potential, adhesion between cells decreases, and thus, expression of β-catenin increases in the cytoplasm and nucleus [5].

**Figure 1:**
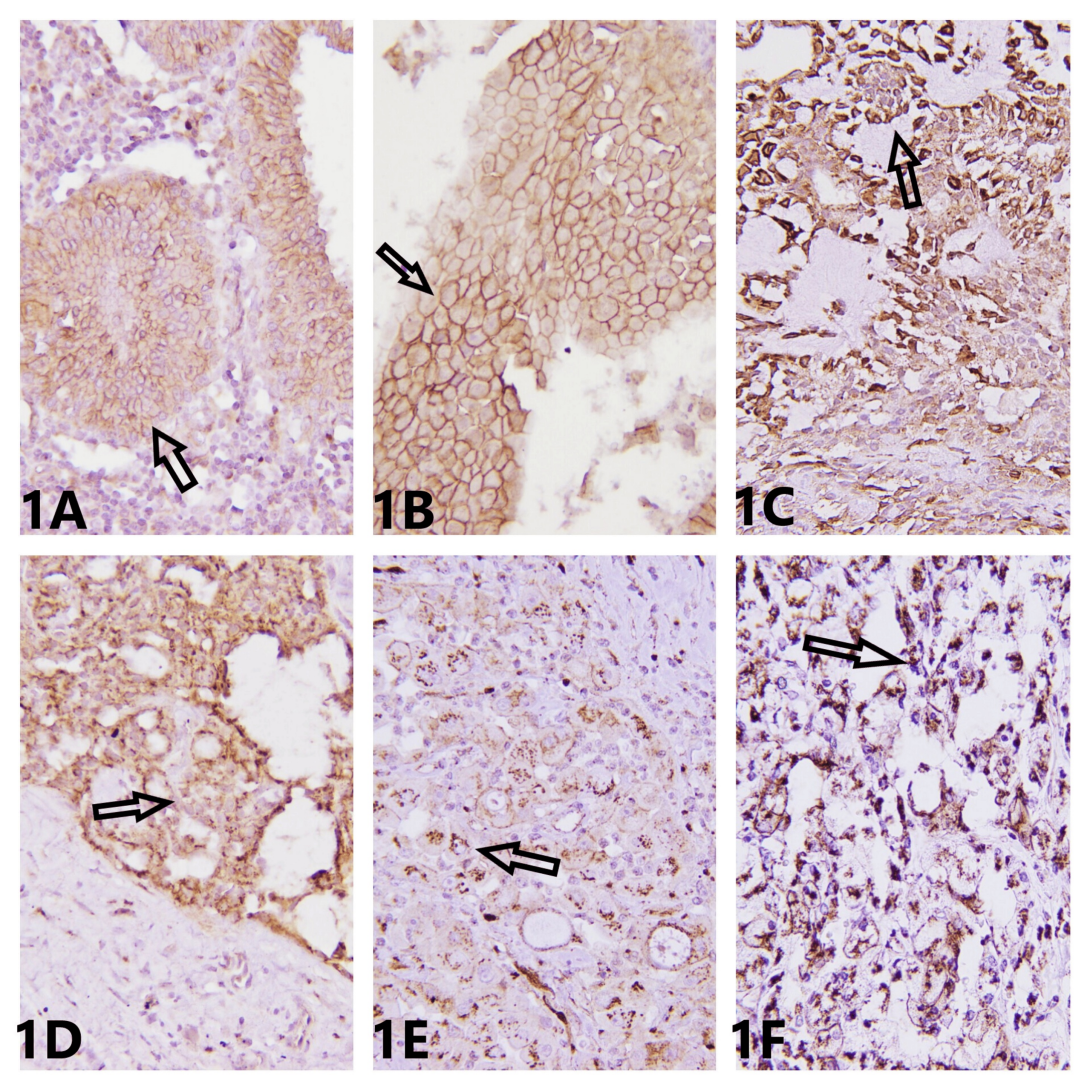
At 400×, mild membranous β-catenin intensity in WT (A), moderate membranous intensity in WT (B), intense membranous intensity in PA (C), mild cytoplasmic intensity in CA ex PA (D), moderate cytoplasmic staining in MEC (E), and intense cytoplasmic staining in ACC (F). WT, Warthin tumor; PA, pleomorphic adenoma; CA ex PA, carcinoma ex pleomorphic adenoma; MEC, mucoepidermoid carcinoma; ACC, acinic cell carcinoma.

In this study, we applied β-catenin to 40 benign cases of salivary glands, including PA, BCA, WT, and CA, and 40 malignant tumors of salivary glands, comprising SDCA, MEC, ACC, ACCA, SCA, and CA ex PA. In our study, it was noted that membranous β-catenin expression was primarily seen in benign tumors and cytoplasmic expression in malignant tumors (Figure 1). WT showed a maximum mean percentage of membranous staining in tumor cells, 92.85%. This finding of membranous expression primarily in benign tumors was in contrast to the study by Akinyamoju et al. [8], which described that both well- and poorly differentiated oral squamous cell carcinoma (SCC) showed membranous expression, while the survey by Yun et al. [9] concurred with our analysis and revealed that 68% of cases of tongue SCC demonstrated partial loss or absence of membranous β-catenin expression in tumor cells. In contrast, a study by Chandrashekar et al. [5] manifested cytoplasmic staining in both benign and malignant tumors, mild in benign and moderate in malignant. A study by Furuse et al. [10] reported increased expression in ductal cells of PA and decreased expression in strands and sheets of PA cells. A survey by Genelhu et al. [11] further enforced that cells lose expression as they acquire invasive properties.

BCA neoplastic cells in our study had a 76.66% membranous pattern, in contrast to a study performed by Kawahara et al. [12], in which all BCA had nuclear β-catenin expression. A single CA case in our study showed 80% membranous staining.

Maximum cytoplasmic staining was viewed in CA ex PA, i.e., 74% in our study, which corresponds with the study carried out by Genelhu et al. [11]. We found only 24% cytoplasmic staining in MEC, which is in concordance with the study by Miguel et al. [13] that showed decreased expression in high-grade MEC while discordant with 80% staining in MEC reported by Chandrashekar et al. [5] and 100% staining in MEC reported by Hakata et al. [14]. The discordance can be due to different study population that need further research studies.

ACCA showed 66% cytoplasmic staining while no nuclear staining was noted, in contrast to a study done by Daa et al. [15], which demonstrated weak membranous staining in almost all cases of ACCA, and two cases showed nuclear staining. Chandrashekar et al. [5] revealed 77% cytoplasmic staining in ACCA, keeping with our study. ACC showed 55% cytoplasmic staining and 40% membranous staining, similar to Yue et al. [16], which depicted low staining in ACC.

Limitations

This study does not correlate β-catenin expression with the histological grade, recurrence or metastatic potential of the malignant tumor. A further limiting factor is that we could not do the follow-up of the patients which demonstrates the prognosis.

## Conclusions

β-catenin is a vital part of epithelial malignancies of the head and neck region, gastrointestinal tract, liver, lung, breast, bladder, and genital tract. Being part of the cadherin-catenin complex, it has a role in cell adhesion in both normal salivary gland and benign salivary gland tumors, thus expressed explicitly in cell membranes. Among benign tumors, WT depicted the most specified localization for membranes. Expression shifted towards the cytoplasm and nucleus in invasive malignancies, highlighting that neoplastic cells lose adherence. Our study suggests that β-catenin could indicate the invasive potential of various salivary gland tumors.
